# Supplementary material for: c-Kit Induces Migration of Triple-Negative Breast Cancer Cells and Is a Promising Target for Tyrosine Kinase Inhibitor Treatment
Source: Int J Mol Sci. 2022 Aug 5;23(15):8702. doi: 10.3390/ijms23158702 (PMC9369219; doi:10.3390/ijms23158702)
Supplement: Supplementary file 1 [file ijms-23-08702-s001.zip › Figure S1.pdf]

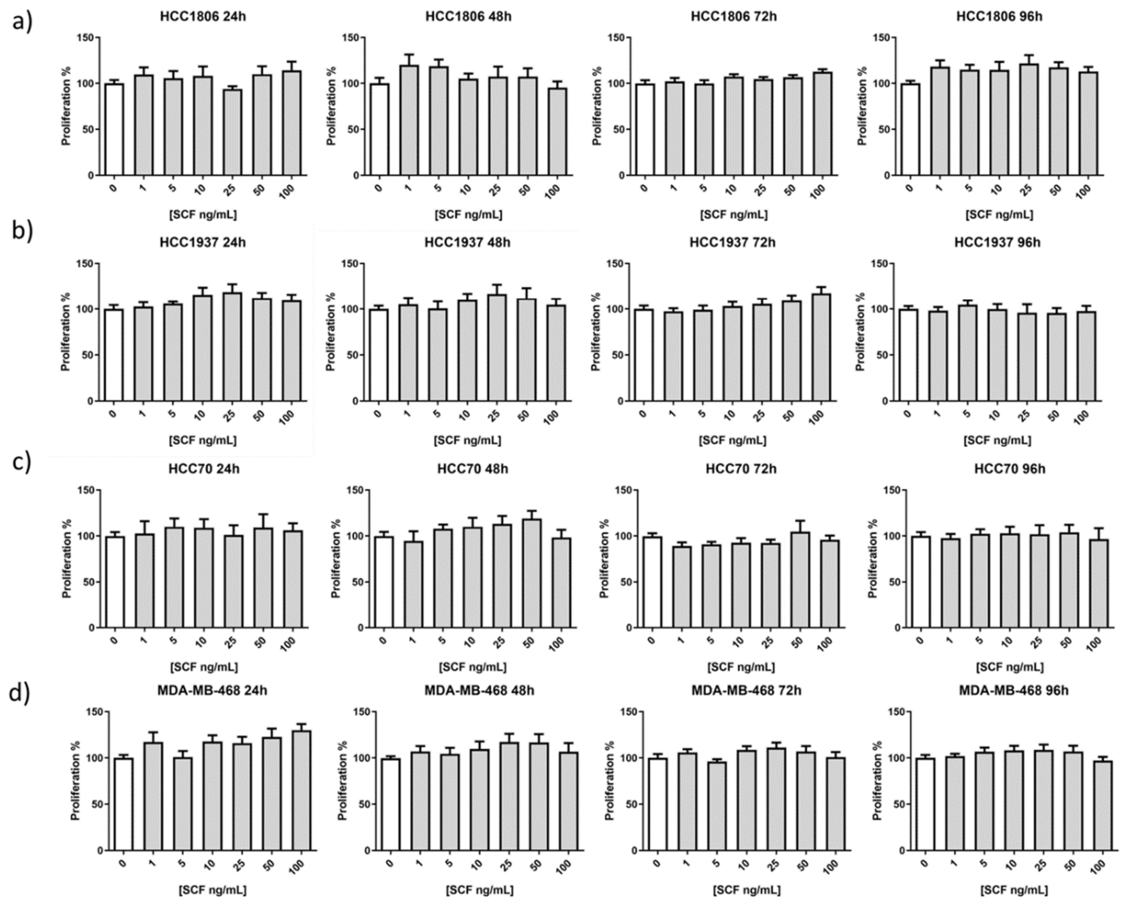

Figure S1. Effect of c-Kit activation on TNBC cell proliferation. HCC-1806 (**a**), HCC-1937 **(b)**, HCC-70 **(c)** and MDA-MB-468 **(d)** cells were incubated with increasing concentrations of SCF as indicated. Cell proliferation was evaluated at 24, 48, 72 and 96 h using the MTT colorimetric assay. Values represent the average of three independent experiments. Error bars indicate the standard error of the mean.
