# Supplementary material for: c-Kit Induces Migration of Triple-Negative Breast Cancer Cells and Is a Promising Target for Tyrosine Kinase Inhibitor Treatment
Source: Int J Mol Sci. 2022 Aug 5;23(15):8702. doi: 10.3390/ijms23158702 (PMC9369219; doi:10.3390/ijms23158702)
Supplement: Supplementary file 1 [file ijms-23-08702-s001.zip › Figure S2.pdf]

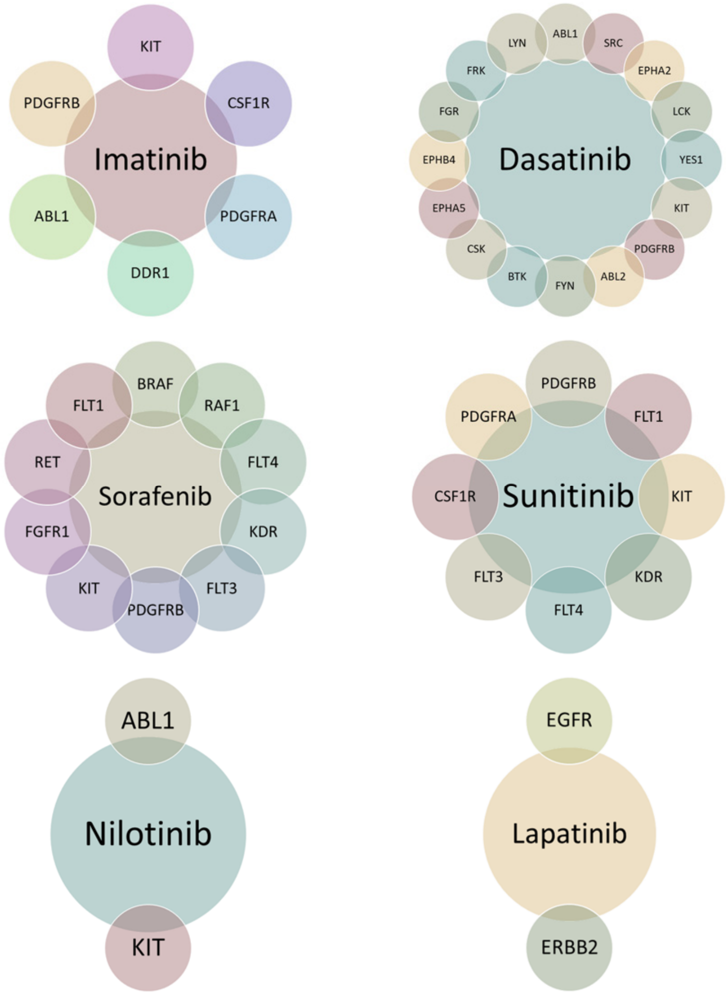

Figure S2. Tyrosine Kinases targeted by selected Tyrosine Kinase Inhibitors. Targeted RTKs and intracellular Tyrosine Kinases for Imatinib, Dasatinib, Sorafenib, Sunitinib, Nilotinib, and Lapatinib are shown.
