# Supplementary material for: c-Kit Induces Migration of Triple-Negative Breast Cancer Cells and Is a Promising Target for Tyrosine Kinase Inhibitor Treatment
Source: Int J Mol Sci. 2022 Aug 5;23(15):8702. doi: 10.3390/ijms23158702 (PMC9369219; doi:10.3390/ijms23158702)
Supplement: Supplementary file 1 [file ijms-23-08702-s001.zip › Figure S3.pdf]

c-Kit

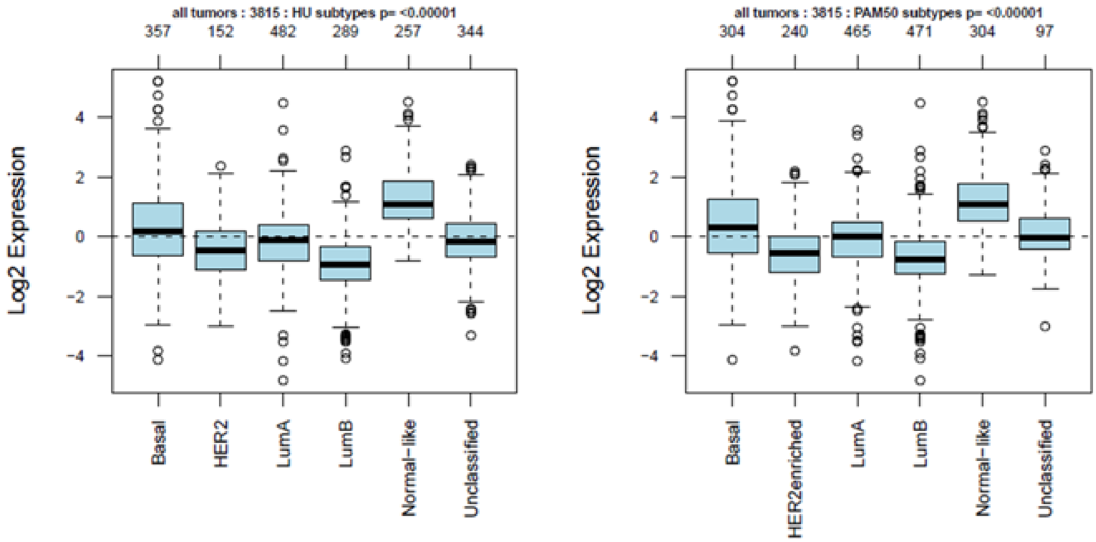
all tumors : 3815 : HU subtypes p= <0.00001
357 152 482 289 257 344
Log2 Expression
Basal
HER2
LumA
LumB
Normal-like
Unclassified
all tumors : 3815 : PAM50 subtypes p= <0.00001
304 240 465 471 304 97
Log2 Expression
Basal
HER2enriched
LumA
LumB
Normal-like
Unclassified

YES

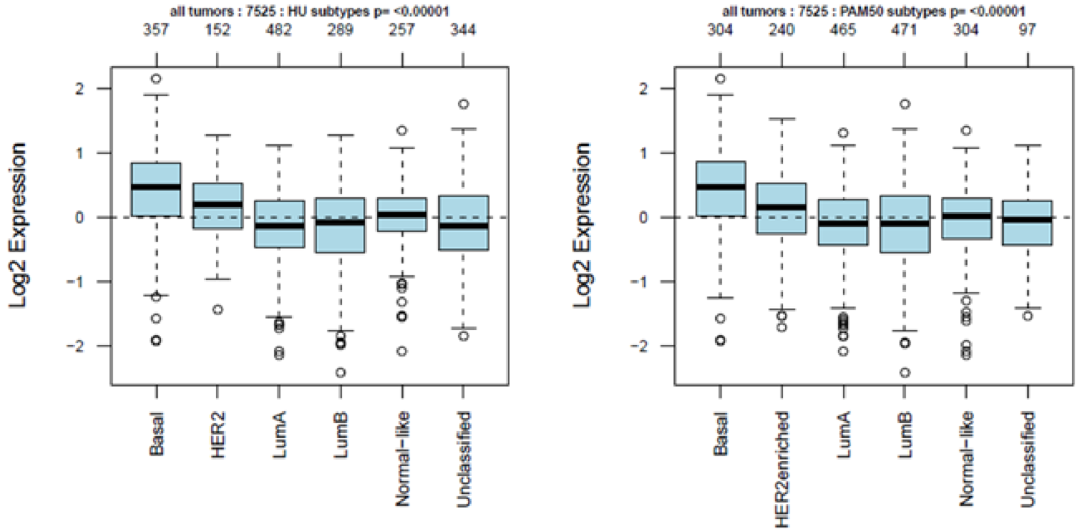
all tumors : 7525 : HU subtypes p= <0.00001
357 152 482 289 257 344
Log2 Expression
Basal
HER2
LumA
LumB
Normal-like
Unclassified
all tumors : 7525 : PAM50 subtypes p= <0.00001
304 240 465 471 304 97
Log2 Expression
Basal
HER2enriched
LumA
LumB
Normal-like
Unclassified

FYN

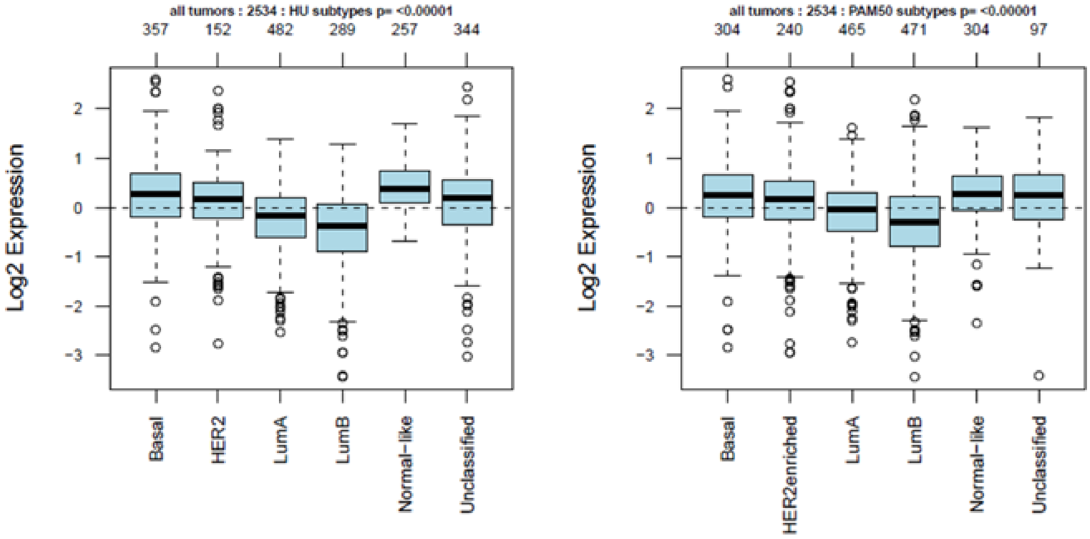
all tumors : 2534 : HU subtypes p= <0.00001
357 152 482 289 257 344
Log2 Expression
Basal
HER2
LumA
LumB
Normal-like
Unclassified
all tumors : 2534 : PAM50 subtypes p= <0.00001
304 240 465 471 304 97
Log2 Expression
Basal
HER2enriched
LumA
LumB
Normal-like
Unclassified

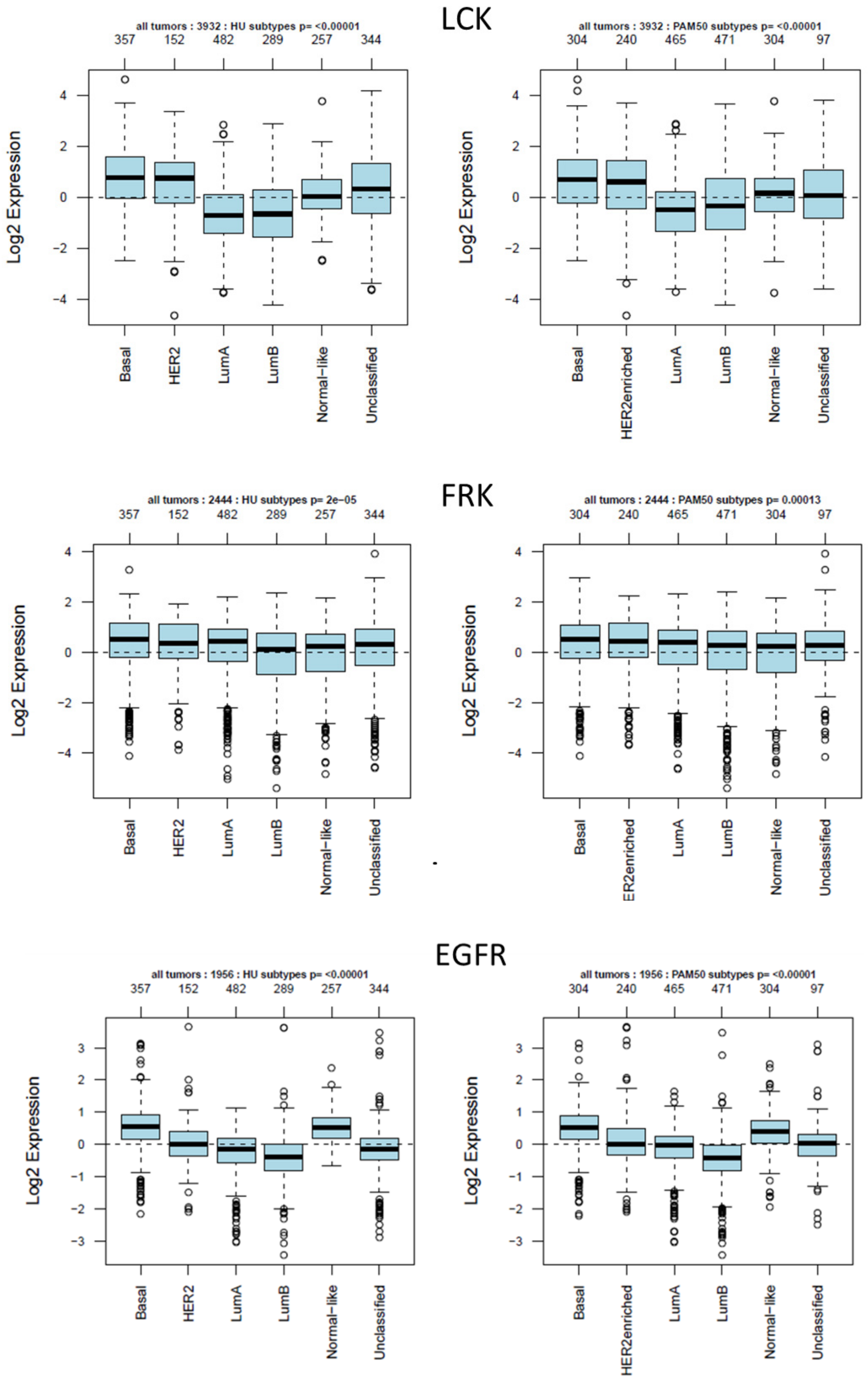
LCK
all tumors : 3932 : HU subtypes p= <0.00001
357 152 482 289 257 344
Log2 Expression
Basal HER2 LumA LumB Normal-like Unclassified
all tumors : 3932 : PAM50 subtypes p= <0.00001
304 240 465 471 304 97
Log2 Expression
Basal HER2enriched LumA LumB Normal-like Unclassified
FRK
all tumors : 2444 : HU subtypes p= 2e-05
357 152 482 289 257 344
Log2 Expression
Basal HER2 LumA LumB Normal-like Unclassified
all tumors : 2444 : PAM50 subtypes p= 0.00013
304 240 465 471 304 97
Log2 Expression
Basal ER2enriched LumA LumB Normal-like Unclassified
EGFR
all tumors : 1956 : HU subtypes p= <0.00001
357 152 482 289 257 344
Log2 Expression
Basal HER2 LumA LumB Normal-like Unclassified
all tumors : 1956 : PAM50 subtypes p= <0.00001
304 240 465 471 304 97
Log2 Expression
Basal HER2enriched LumA LumB Normal-like Unclassified

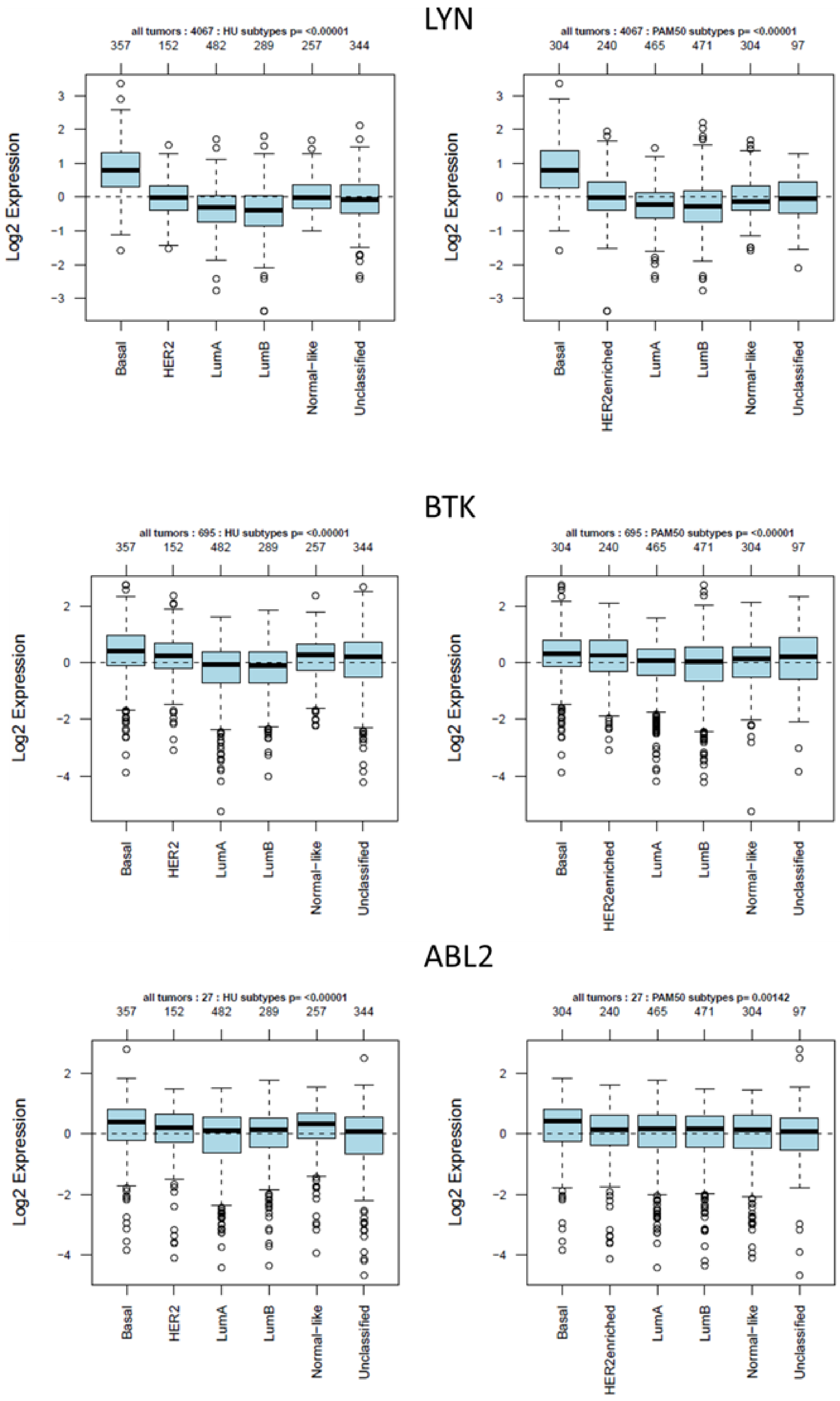
LYN
all tumors : 4067 : HU subtypes p= <0.00001
357 152 482 289 257 344
Log2 Expression
Basal
HER2
LumA
LumB
Normal-like
Unclassified
all tumors : 4067 : PAM50 subtypes p= <0.00001
304 240 465 471 304 97
Log2 Expression
Basal
HER2enriched
LumA
LumB
Normal-like
Unclassified
BTK
all tumors : 695 : HU subtypes p= <0.00001
357 152 482 289 257 344
Log2 Expression
Basal
HER2
LumA
LumB
Normal-like
Unclassified
all tumors : 695 : PAM50 subtypes p= <0.00001
304 240 465 471 304 97
Log2 Expression
Basal
HER2enriched
LumA
LumB
Normal-like
Unclassified
ABL2
all tumors : 27 : HU subtypes p= <0.00001
357 152 482 289 257 344
Log2 Expression
Basal
HER2
LumA
LumB
Normal-like
Unclassified
all tumors : 27 : PAM50 subtypes p= 0.00142
304 240 465 471 304 97
Log2 Expression
Basal
HER2enriched
LumA
LumB
Normal-like
Unclassified

Figure S3. Expression of Receptor Tyrosine Kinases (RTKs) and intracellular tyrosine kinases in Breast Cancer. Expression of RTKs and intracellular tyrosine kinases targeted by Imatinib, Dasatinib, Sorafenib, Sunitinib, Nilotinib, and Lapatinib was explored using data from the Gene expression-based Outcome for Breast cancer Online (GOBO) tool. Differential expression of RTKs and intracellular tyrosine kinases among the basal, HER2-enriched, normal-like, and unclassified subgroups is shown. RTKs and intracellular tyrosine kinases showing significantly higher levels of expression in the basal subtype (LCK, YES, FYN, FRK, LYN, BTK, ABL, and c-Kit) were selected for further analysis.
